# Exposure to the saturated free fatty acid palmitate alters microglia inflammatory response

**DOI:** 10.1186/1750-1326-8-S1-P42

**Published:** 2013-09-13

**Authors:** Linda Tracy, Filip Bergqvist, Elena Ivanova, Kristin Jacobsen, Kerstin Iverfeldt

**Affiliations:** 1 Dept. of Neurochemistry Stockholm University, Stockholm, Sweden

## Background

High calorie diets with increased proportion of saturated fats, together with lack of exercise, are contributing to a growing number of obese and diabetic people in the world. It has been shown that obesity and type 2 diabetes increase the risk for developing dementia and Alzheimer’s disease [1]. Further, a correlation between high fat diets and impaired cognitive function has been proposed [2]. In addition, obesity is also linked to a chronic low grade systemic inflammation [3]. High fat diets have been shown to cause neuroinflammation [4], and neuroinflammation is in turn linked to neurodegeneration [5]. Microglia, the macrophages of the brain, have the ability to respond to environmental changes, and to display diverse phenotypes depending on the stimuli. Activated microglia are believed to play different roles that may either be neuroprotective or promote neurodegeneration [6]. It is therefore of importance to elucidate what effects free fatty acids (FFAs) have on microglia and how they affect microglia challenged with inflammatory stimuli.

## Results

Our results indicate that the saturated FFA palmitate on its own induces alternative activation of BV-2 microglia cells. Further, pre-exposure to palmitate changed the response of microglia to lipopolysaccharide (LPS). We show that palmitate affects the mRNA levels of the pro-inflammatory cytokines interleukin-1β and interleukin-6. The transcription factor CCAAT/enhancer binding protein δ is also affected by pre-exposure to palmitate. Furthermore, the phagocytic activity of microglia was investigated using fluorescent beads. By analyzing the bead up-take by fluorescence activated cell sorting (FACS) we found that palmitate alone, as well as together with LPS, stimulated the phagocytic activity of microglia.

## Conclusion

Thus, exposure of microglia to increased levels of free fatty acids may alter the consequences of classical inflammatory stimuli.
